# Increased INR Values Predict Accelerating Deterioration and High Short-Term Mortality Among Patients Hospitalized With Cirrhosis or Advanced Fibrosis

**DOI:** 10.3389/fmed.2021.762291

**Published:** 2021-11-18

**Authors:** Ying Wang, Fuchen Dong, Shuning Sun, Xianbo Wang, Xin Zheng, Yan Huang, Beiling Li, Yanhang Gao, Zhiping Qian, Feng Liu, Xiaobo Lu, Junping Liu, Haotang Ren, Yubao Zheng, Huadong Yan, Guohong Deng, Liang Qiao, Yan Zhang, Wenyi Gu, Xiaomei Xiang, Yi Zhou, Baoyan Xu, Yixin Hou, Qun Zhang, Yan Xiong, Congcong Zou, Jun Chen, Zebing Huang, Xiuhua Jiang, Tingting Qi, Sen Luo, Yuanyuan Chen, Na Gao, Chunyan Liu, Wei Yuan, Xue Mei, Jing Li, Tao Li, Rongjiong Zheng, Xinyi Zhou, Weituo Zhang, Hai Li, Zhongji Meng

**Affiliations:** ^1^Department of Infectious Disease, Hubei Clinical Research Center for Precise Diagnosis and Treatment of Liver Cancer, Taihe Hospital, Hubei University of Medicine, Shiyan, China; ^2^Chinese Chronic Liver Failure Consortium, China; ^3^Department of Gastroenterology, School of Medicine, Ren Ji Hospital, Shanghai Jiao Tong University, Shanghai, China; ^4^Department of Infectious Diseases, Southwest Hospital, Third Military Medical University (Army Medical University), Chongqing, China; ^5^Center of Integrative Medicine, Beijing Ditan Hospital, Capital Medical University, Beijing, China; ^6^Department of Infectious Diseases, Tongji Medical College, Institute of Infection and Immunology, Union Hospital, Huazhong University of Science and Technology, Wuhan, China; ^7^Hunan Key Laboratory of Viral Hepatitis, Department of Infectious Diseases, Xiangya Hospital, Central South University, Changsha, China; ^8^Hepatology Unit, Department of Infectious Diseases, Nanfang Hospital, Southern Medical University, Guangzhou, China; ^9^Department of Hepatology, The First Hospital of Jilin University, Jilin, China; ^10^Department of Liver Intensive Care Unit, Shanghai Public Health Clinical Centre, Fudan University, Shanghai, China; ^11^Department of Infectious Diseases and Hepatology, The Second Hospital of Shandong University, Jinan, China; ^12^Infectious Disease Center, The First Affiliated Hospital of Xinjiang Medical University, Urumqi, China; ^13^Department of Infectious Diseases, Henan Provincial People's Hospital, Henan, China; ^14^State Key Laboratory for Diagnosis and Treatment of Infectious Diseases, Collaborative Innovation Center for Diagnosis and Treatment of Infectious Disease, The First Affiliated Hospital, Zhejiang University School of Medicine, Hangzhou, China; ^15^Department of Infectious Diseases, The Third Affiliated Hospital, Sun Yat-sen University, Guangzhou, China; ^16^Department of Infectious Diseases, Hwamei Hospital, Ningbo No. 2 Hospital, University of Chinese Academy of Sciences, Ningbo, China; ^17^Clinical Research Center, Shanghai Jiao Tong University School of Medicine, Shanghai, China

**Keywords:** acute on chronic liver failure (ACLF), cirrhosis, advanced fibrosis, international normalized ratio (INR), short-term prognosis

## Abstract

**Background and Objective:** An increase in the international normalized ratio (INR) is associated with increased mortality in patients with cirrhosis and other chronic liver diseases, while little is known about the quantitative relationship. This study aimed to investigate the quantitative relationship between the INR and short-term prognosis among patients hospitalized with cirrhosis or advanced fibrosis and to evaluate the role of the INR as a risk factor for short-term liver transplant (LT)-free mortality in these patients.

**Patients and Methods:** This study prospectively analyzed multicenter cohorts established by the Chinese Acute-on-Chronic Liver Failure (CATCH-LIFE) study. Cox regression was used to describe the relationship between the INR and independent risk factors for short-term LT-free mortality. Forest plots were used in the subgroup analysis. Generalized additive models (GAMs) and splines were used to illustrate the quantitative curve relationship between the INR and the outcome and inflection point on the curve.

**Results:** A total of 2,567 patients with cirrhosis and 924 patients with advanced fibrosis were included in the study. The 90-day LT-free mortality of patients with cirrhosis and advanced fibrosis was 16.7% (428/2,567) and 7.5% (69/924), respectively. In the multivariable Cox regression analysis, the increase in the INR was independently associated with the risk of 90-day LT-free mortality both in patients with cirrhosis (HR, 1.06; 95% CI, 1.04–1.07, *p* < 0.001) and in patients with advanced fibrosis (HR, 1.09; 95% CI, 1.06–1.12, *p* < 0.001). An INR of 1.6/1.7 was found to be the starting point of coagulation dysfunction with a rapid increase in mortality in patients with cirrhosis or in patients with advanced fibrosis, respectively. A 28-day LT-free mortality of 15% was associated with an INR value of 2.1 in both cirrhosis and advanced fibrosis patients.

**Conclusions:** This study was the first to quantitatively describe the relationship between the INR and short-term LT-free mortality in patients with cirrhosis or advanced fibrosis. The starting points of INR indicating the rapid increase in mortality and the unified cutoff value of coagulation failure in cirrhosis and advanced fibrosis, will help clinicians accurately recognize early disease deterioration.

## Introduction

Cirrhosis and other chronic liver diseases are the main causes of death, affecting 1.5 billion people worldwide (1, 2) and accounting for 1.3 million deaths every year (3). Most chronic liver disease patients will remain in a stable state, while upon acute liver injury (ALI), they may progress to acute decompensation or even organ failure; the latter is defined as acute-on-chronic liver failure (ACLF) and is characterized by a high short-term mortality rate of over 50% (4, 5).

Most patients with liver cirrhosis or other chronic liver diseases hospitalized with ALI have coagulation disorders, which are associated with significantly prolonged prothrombin time (PT) and an increased international normalized ratio (INR) (6). As an indicator of severe liver injury, the INR has been included in the diagnostic criteria of ACLF by the European Association for The Study of the Liver (EASL) and the Asian-Pacific Association for the Study of the Liver (APASL) (7–9). The INR has also been incorporated in scoring systems such as the model for end-stage liver disease (MELD), MELD-Na, and chronic liver failure-sequential organ failure assessment (CLIF-SOFA) for assessing the severity of cirrhosis and liver failure (10–12).

However, the cutoff value of the INR for the diagnosis of ACLF has long been controversial in the East and the West (13). The APASL considers that an INR ≥ 1.5 and a total bilirubin (TB) ≥ 5 mg/dL in patients with cirrhosis and noncirrhotic chronic liver disease were important indicators for the diagnosis of ACLF. The INR cutoff value used by the APASL in the diagnostic criteria of ACLF is based on the definition of acute liver failure (9, 14–16). The EASL considers an INR ≥2.5 in patients with cirrhosis as the cutoff value for coagulation failure in the diagnostic criteria of ACLF (7). It is unclear whether coagulation failure in cirrhosis and advanced fibrosis can share the same cutoff value for the INR. Therefore, it is pivotal to explore the evidence-based cutoff values of the INR for coagulation failure in patients with cirrhosis and advanced fibrosis, and this is important to unify the thresholds of coagulation failure among the East and West.

This study was based on a large, multicenter, prospective cohort of patients in areas where hepatitis B virus (HBV) is highly endemic, and this study included both patients with cirrhosis and patients with advanced fibrosis (17, 18). This study aimed to investigate the quantitative relationship between the INR and the short-term (28 /90-day) LT-free mortality in patients with cirrhosis and advanced fibrosis separately and to provide evidence for establishing a reliable INR cutoff value for the diagnosis of coagulation failure.

## Patients and Methods

### Patients

Patients with cirrhosis and other chronic liver diseases (3) hospitalized for acute decompensation (AD) (7) or acute liver injury (ALI) were enrolled in the Chinese Acute-on Chronic Liver Failure (CATCH-LIFE) study cohorts (NCT02457637 and NCT03641872) from 16 Chinese tertiary hospitals during the periods of January 2015–December 2016 and January 2018–December 2019 (17–19). Detailed inclusion and exclusion criteria have been published elsewhere (17–19). This study was approved by the Ethics Committee of Renji Hospital (the leading center of the CATCH-LIFE study), School of Medicine, Shanghai Jiao Tong University, Shanghai, China. Signed informed consent was obtained from all patients.

Cirrhosis was diagnosed based on computed tomography/magnetic resonance imaging, laboratory examination, and clinical symptoms (20). Cirrhosis without prior AD was defined as cirrhotic patients who developed AD for the first time (7). Those with a prior history of AD were defined as cirrhotic patients with prior (one or more episodes) AD (7). Advanced fibrosis was defined as noncirrhotic patients who had a chronic liver disease history of at least 6 months and had FIB-4 scores over 1.45 (21).

**Figure 1:**
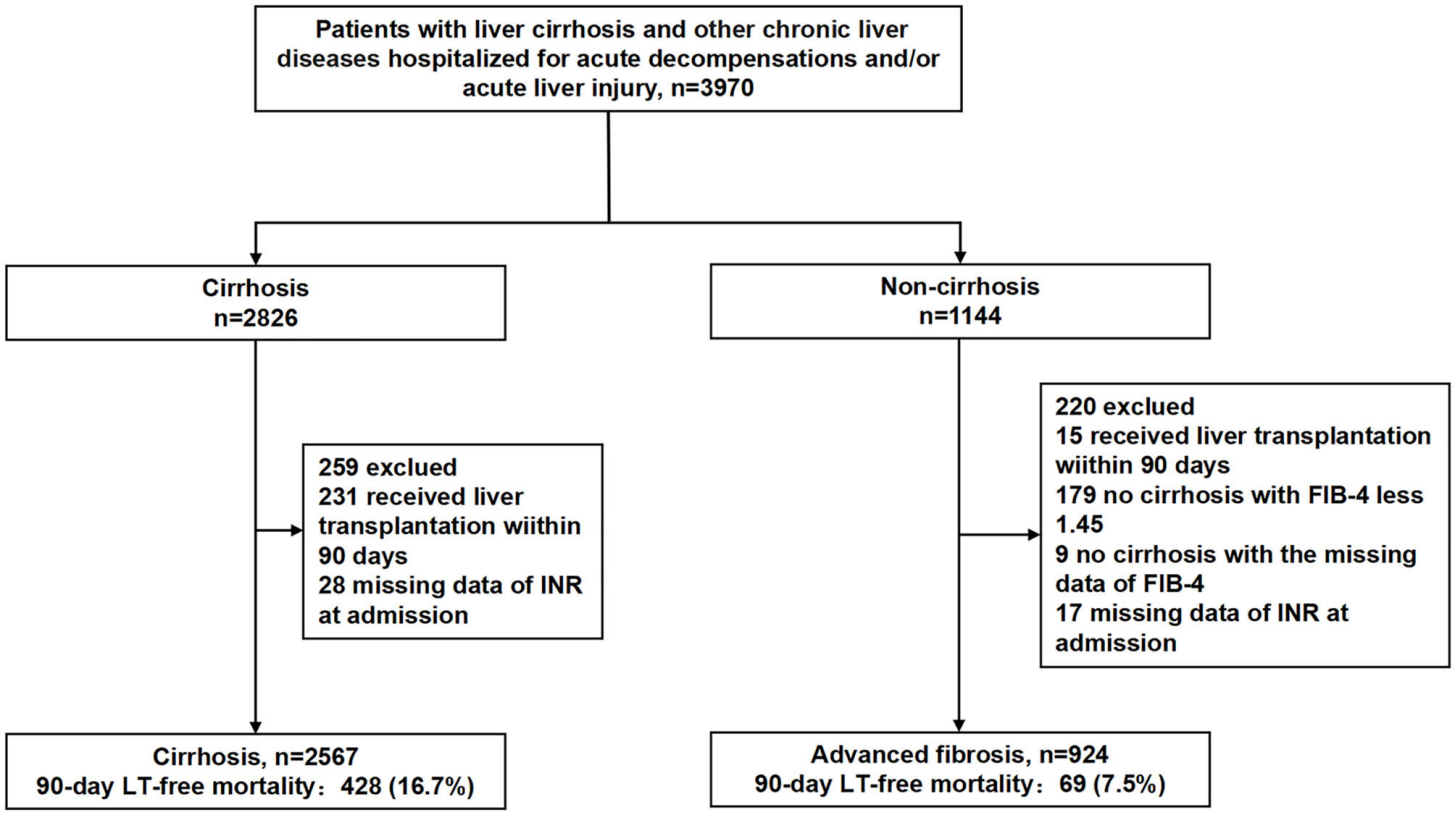
Study participants. FIB-4, fibrosis-4 index; INR, international normalized ratio; LT, liver transplantation.

Among the cirrhotic patients and the patients with advanced fibrosis, those who received liver transplant within 90 days, those with INR values missing values on admission, patients with FIB-4 ≤ 1.45 and patients with missing FIB-4 values in noncirrhotic patients were not included in the analysis (Figure 1; Supplementary Figure 1).

### Data Collection

The following demographic and clinical information was collected on admission: age, sex, etiology of the liver disease, acute decompensated events, laboratory parameters, clinical symptoms involved in Child-Turcotte-Pugh [CTP] (22), and CLIF-SOFA scores. Details about the data collected in this study can be found elsewhere (17, 18).

### Outcomes

The primary and secondary endpoints of the study were 90 and 28-day liver transplant (LT)-free mortality, respectively.

### Statistical Analysis

Data are presented as medians and the first and third quartiles for continuous variables and as frequencies (%) for categorical variables at baseline. A multivariate Cox proportional hazard (COXPH) model was used to analyze the correlation between the INR and 90-day LT-free mortality. Important risk factors and potential confounding factors were adjusted, and these included age, sex, and etiology of underlying chronic liver disease, overt ascites, gastrointestinal bleeding, bacterial infection, hepatic encephalopathy (HE) grades, TB and creatinine. The risk of 90-day LT-free mortality was expressed by continuous variables as the risk ratio (HR), which was calculated by each unit of the INR. In patients with cirrhosis, the INR was categorized into fine levels (<1.2, 1.2–1.5, 1.5–2.0, 2–2.5, and ≥2.5), and patients with an INR <1.2 were used as a reference. In advanced fibrosis, the INR was categorized into 4 levels (<1.5, 1.5–2.0, 2.0–2.5, and >2.5), and patients with an INR <1.5 were used as a reference. Forest plots were used for subgroup analysis.

The nonlinear relationship between the INR and 90-day mortality was plotted as an “INR-mortality correlation curve”. The estimated mortality rates corresponding to the INR values in the curves and the independent effect of INR on mortality were shown by the confounding factors adjusted generalized additive model (GAM) (23). Spline (24) was used as a connecting function to select the GAM and smoothing parameters to optimize the Akaike information criterion. The second derivative of the INR to mortality was used to describe the nonlinear relationship (to obtain the maximum acceleration peak). The maximum acceleration point on the GAM curve was defined as the starting point of the INR for disease deterioration. Meanwhile, the INR value corresponding to 15% LT-free mortality within 28 days was considered the clinical cutoff value for coagulation failure (the definition from the CANONIC study) (7). Statistical analyses were performed using R version 3.1.2 (R Foundation for Statistical Computing, Vienna, Austria) and MATLAB 2016b. A two-sided *p* < 0.05 was considered statistically significant.

## Results

### Characteristics of the Patients

**Figure 2:**
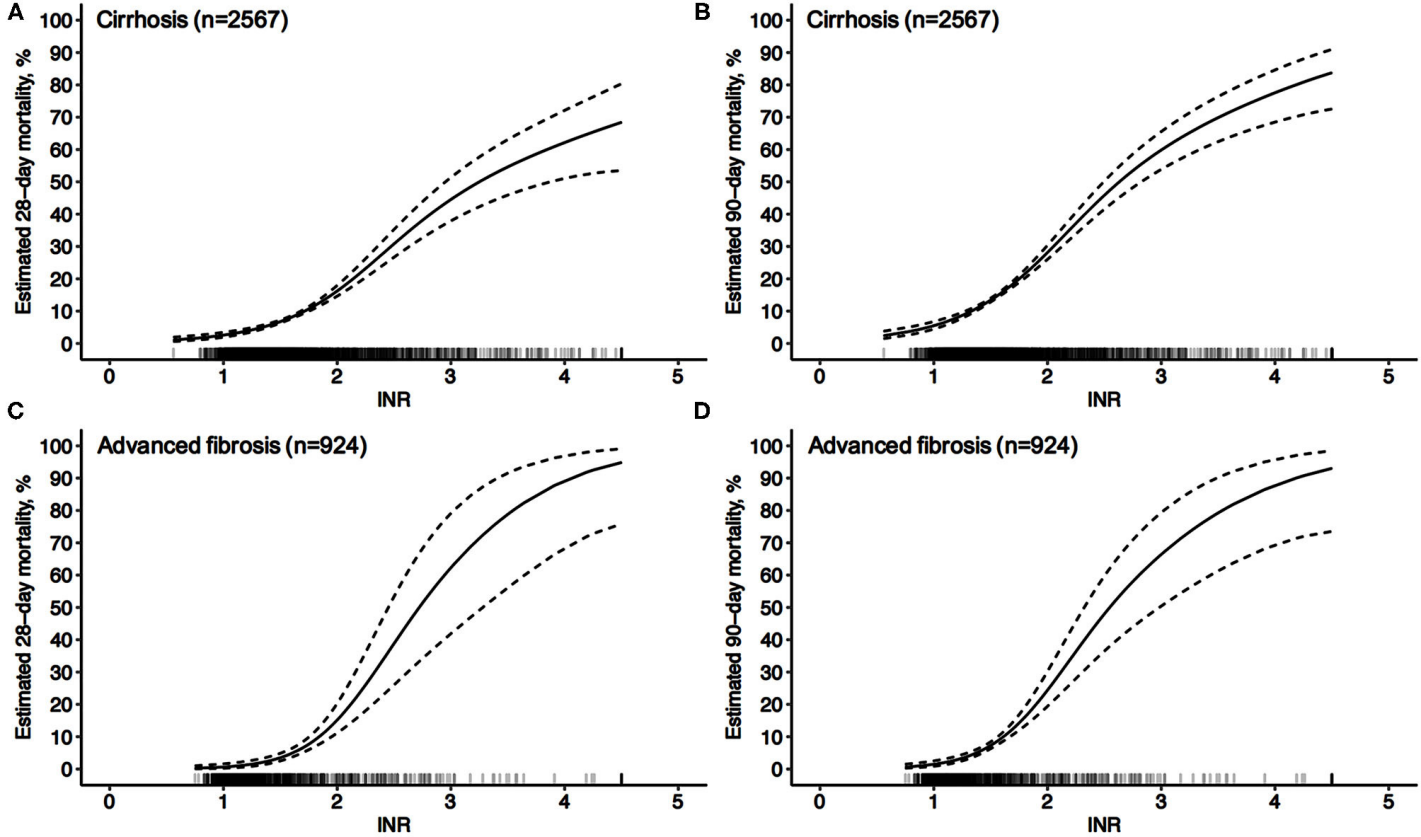
Unadjusted probability of LT-free mortality by baseline INR in cirrhosis and advanced fibrosis. **(A,B)** 28-day **(A)** and 90-day **(B)** LT-free mortality by baseline INR in cirrhosis, **(C,D)** 28-day **(C)** and 90-day **(D)** LT-free mortality by baseline INR in advanced fibrosis.

As shown in Figure 1, 2,567 patients with cirrhosis and 924 patients with advanced fibrosis were ultimately included in the analysis. The 90-day LT-free mortality of cirrhosis and advanced fibrosis were 16.7% (428/2,567) and 7.5% (69/924), respectively. In Figure 2, we depict the uncorrected INR in relation to the 28/90-day LT-free mortality using GAM. In cirrhotic patients and advanced fibrosis, as the INR increased, the disease severity (MELD score, CLIF-SOFA score, and Child Pugh score) and the corresponding short-term LT-free mortality increased (Tables 1, 2).

**Table 1:** Baseline characteristics of patients with cirrhosis based on different INR groups at admission.

**Characteristics**	**INR <1.2**	**1.2 ≤ INR <1.5**	**1.5 ≤ INR <2.0**	**2.0 ≤ INR <2.5**	**INR ≥ 2.5**
	***n* = 468**	***n* = 852**	***n* = 724**	***n* = 275**	***n* = 248**
**Demographic**					
Age	54 [47,62]	53 [45,61]	50 [44,58]	49 [42,57]	51 [42,59]
Gender	287 (61.3)	609 (71.5)	562 (77.6)	218 (79.3)	197 (79.4)
**Etiology**					
Alcoholic	55 (11.8)	117 (13.7)	88 (12.2)	34 (12.4)	23 (9.3)
HBV	238 (50.9)	528 (62.0)	531 (73.3)	212 (77.1)	203 (81.9)
AIH	93 (19.9)	90 (10.6)	48 (6.6)	6 (2.2)	10 (4.0)
Others	82 (17.5)	117 (13.7)	57 (7.9)	23 (8.4)	12 (4.8)
**Acute decompensation**					
**HE**					
Grade0	446 (95.3)	774 (90.8)	645 (89.1)	239 (86.9)	187 (75.4)
Grade1	6 (1.3)	33 (3.9)	35 (4.8)	16 (5.8)	13 (5.2)
Grade2	14 (3.0)	31 (3.6)	31 (4.3)	13 (4.7)	30 (12.1)
Grade3	1 (0.2)	11 (1.3)	10 (1.4)	4 (1.5)	13 (5.2)
Grade4	1 (0.2)	3 (0.4)	3 (0.4)	3 (1.1)	5 (2.0)
Infection	71 (15.2)	203 (23.8)	218 (30.1)	86 (31.3)	112 (45.2)
Ascites	215 (45.9)	502 (58.9)	483 (66.7)	205 (74.5)	187 (75.4)
GI bleeding	113 (24.1)	217 (25.5)	131 (18.1)	32 (11.6)	15 (6.0)
**Laboratory tests**					
TB	1.5 [0.9,3.1]	2.2 [1.2,5.4]	5.0 [2.5,13.5]	13.6 [6.7,22.6]	20.8 [13.0,29.3]
INR	1.1 [1.0,1.2]	1.3 [1.3,1.4]	1.7 [1.6,1.8]	2.2 [2.1,2.4]	3.0 [2.7,3.5]
Cr	0.8 [0.6,0.9]	0.8 [0.6,1.0]	0.8 [0.6,1.0]	0.8 [0.7,1.0]	0.9 [0.7,1.2]
**Scores**					
MELD	9 [7,12]	12 [10,16]	18 [15,23]	25 [22,28]	31 [28,34]
CLIF-SOFA	2 [1,3]	4 [3,5]	6 [5,7]	7 [6,8]	7 [7,9]
Child-Pugh	8 [6,9]	8 [7,1]	10 [9,11]	11 [10,12]	12 [11,13]
**Outcome**					
28-day LT-free mortality	10 (2.1)	29 (3.4)	44 (6.1)	55 (20.0)	98 (39.5)
90-day LT-free mortality	23 (4.9)	65 (7.6)	105 (14.5)	98 (35.6)	137 (55.2)

*HBV, hepatitis B virus; AIH, autoimmune hepatitis; HE, hepatic encephalopathy; GI bleeding, gastrointestinal bleeding; TB, total bilirubin; INR, international normalized ratio; Cr, creatinine; MELD, model for end-stage liver disease; CLIF-SOFA, chronic liver failure-sequential organ failure assessment; LT, liver transplantation*.

**Table 2:** Baseline characteristics of patients with advanced fibrosis based on different INR groups at admission.

**Characteristics**	**INR <1.2**	**1.2 ≤ INR <1.5**	**1.5 ≤ INR <2.0**	**2.0 ≤ INR <2.5**	**INR ≥ 2.5**
	***n* = 384**	***n* = 242**	***n* = 165**	***n* = 75**	***n* = 85**
**Demographic**					
Age	44 [35,53]	41 [33,49]	43 [34,50]	40 [34,49]	39 [37,42]
Gender	263 (68.5)	194 (80.2)	134 (81.2)	58 (77.3)	47 (81.0)
**Etiology**					
Alcoholic	14 (3.6)	10 (4.1)	3 (1.8)	1 (1.3)	3 (5.2)
HBV	278 (72.4)	213 (88.0)	153 (92.7)	74 (98.7)	52 (89.7)
AIH	45 (11.7)	9 (3.7)	6 (3.6)	0 (0.0)	1 (1.7)
Others	47 (12.2)	10 (4.1)	3 (1.8)	0 (0.0)	2 (3.4)
**Acute decompensation**					
**HE**					
Grade0	382 (99.5)	239 (98.8)	160 (97.0)	70 (93.3)	37 (63.8)
Grade1	2 (0.5)	3 (1.2)	3 (1.8)	4 (5.3)	4 (6.9)
Grade2	0 (0.0)	0 (0.0)	1 (0.6)	0 (0.0)	9 (15.5)
Grade3	0 (0.0)	0 (0.0)	0 (0.0)	0 (0.0)	7 (12.1)
Grade4	0 (0.0)	0 (0.0)	1 (0.6)	1 (1.3)	1 (1.7)
Infection	23 (6.0)	20 (8.3)	24 (14.5)	21 (28.0)	19 (32.8)
Ascites	12 (3.1)	19 (7.9)	36 (21.8)	26 (34.7)	26 (44.8)
GI bleeding	1 (0.3)	0 (0.0)	0 (0.0)	0 (0.0)	0 (0.0)
**Laboratory tests**					
TB	2.3 [1.2,7.2]	5.8 [2.2,12.8]	12.1 [7.2,19.4]	16.4 [12.3,23.7]	21.8 [15.4,25.9]
INR	1.1 [1.0,1.1]	1.3 [1.3,1.4]	1.7 [1.6,1.8]	2.2 [2.1,2.4]	2.9 [2.7,3.6]
Cr	0.8 [0.6,0.9]	0.8 [0.6,0.9]	0.8 [0.6,0.9]	0.8 [0.6,0.9]	0.8 [0.7,1.0]
**Scores**					
MELD	10 [8,14]	16 [12,19]	22 [2,24]	26 [25,27]	35 [28,36]
CLIF-SOFA	2 [1,3]	4 [3,6]	7 [6,7]	7 [7,7]	8 [7,9]
Child-Pugh	6 [5,7]	7 [6,8]	8 [8,9]	10 [9,11]	12 [12,13]
**Outcome**					
28-day LT-free mortality	0 (0.0)	1 (0.4)	4 (2.4)	11 (14.7)	24 (41.4)
90-day LT-free mortality	0 (0.0)	5 (2.1)	12 (7.3)	23 (30.7)	29 (50.0)

*HBV, hepatitis B virus; AIH, autoimmune hepatitis; HE, hepatic encephalopathy; GI bleeding, gastrointestinal bleeding; TB, total bilirubin; INR, international normalized ratio; Cr, creatinine; MELD, model for end-stage liver disease; CLIF-SOFA, chronic liver failure-sequential organ failure assessment; LT, liver transplantation*.

### INR Is an Independent Risk Factor for 90-Day LT-Free Mortality

**Figure 3:**
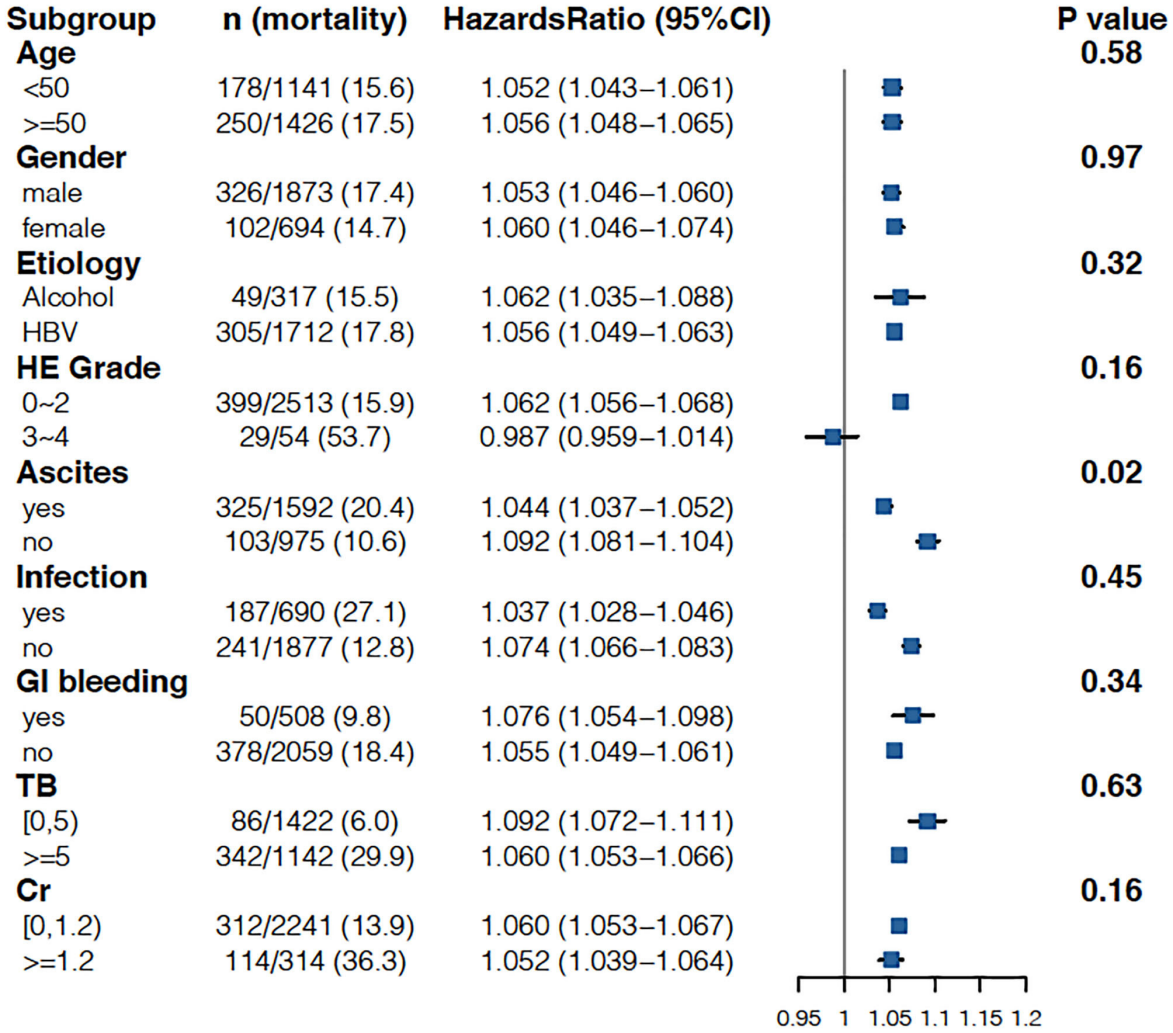
Stratified analyses of adjusted risk of 90-day LT-free mortality in cirrhosis patients according to strata of baseline covariates INR. HBV, hepatitis B virus; HE, hepatic encephalopathy; GI bleeding, gastrointestinal bleeding; TB, total bilirubin; Cr, creatinine; INR, international normalized ratio.

**Figure 4:**
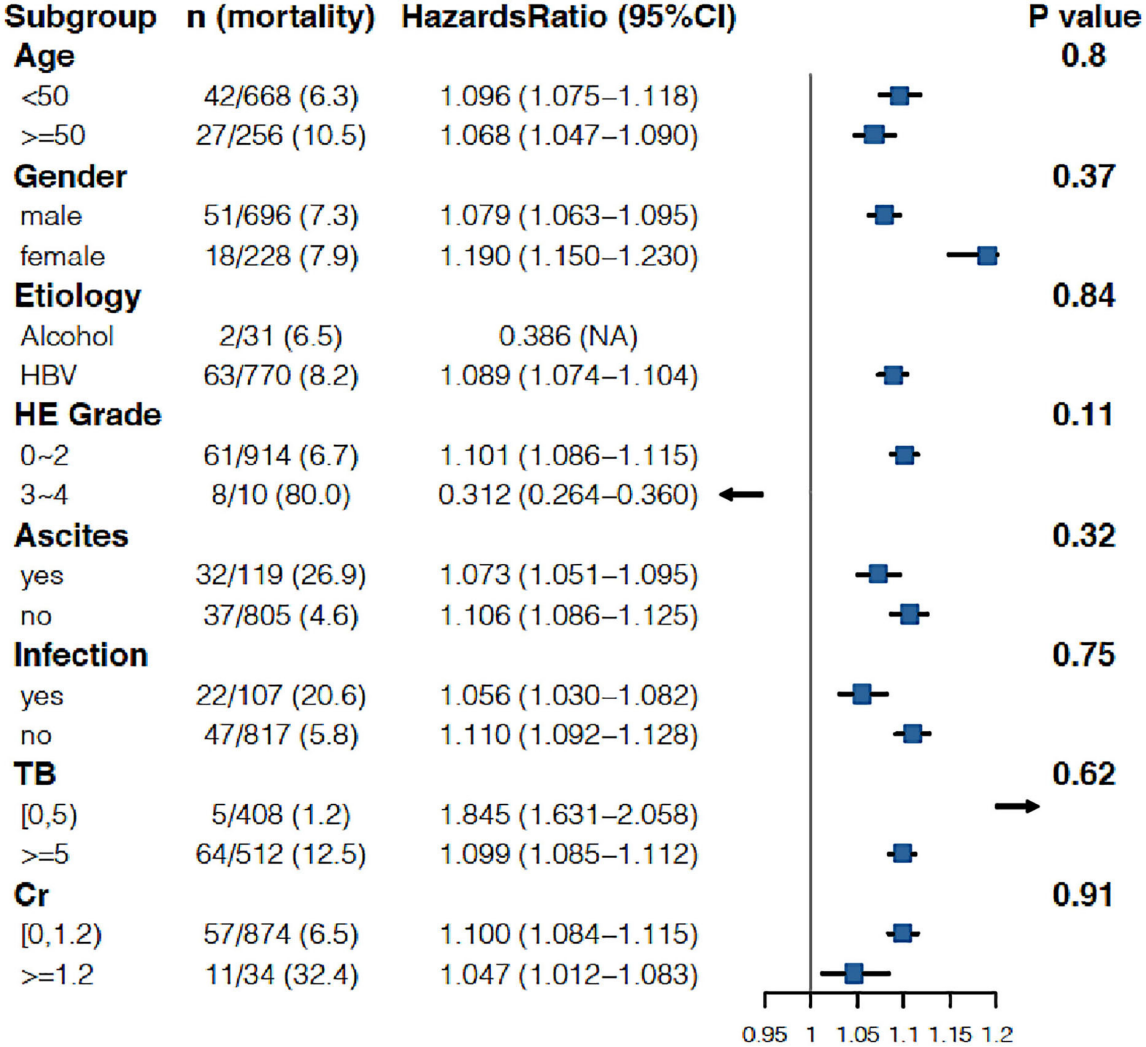
Stratified analyses of adjusted risk of 90-day LT-free mortality in advanced fibrosis patients according to strata of baseline covariates INR. HBV, hepatitis B virus; HE, hepatic encephalopathy; GI bleeding, gastrointestinal bleeding; TB, total bilirubin; Cr, creatinine; INR, international normalized ratio.

As shown in Table 3, when the INR was used as a continuous variable, it was an independent risk factor for 90-day LT-free mortality in both cirrhotic and advanced fibrosis patients (Table 3). The analysis of the categorical variables showed that when the INR > 1.5, the elevated INR had a significantly greater impact on mortality than patients with an INR <1.5 in both cirrhosis and advanced fibrosis patients. The INR was an independent risk factor for 90-day LT-free mortality without the interaction of most indicators except hepatic encephalopathy 3~4, a phenomenon existed that was consistent in patients with cirrhosis and advanced fibrosis (Figures 3, 4). The results of the 28-day univariate and multivariable analyses are also shown in Supplementary Table 1.

**Table 3:** The unadjusted and adjusted hazard ratios of INR for 90-day transplantation-free mortality in patients with cirrhosis and advanced fibrosis.

**INR**	***n***	**Death (%)**	**HR, 95% CI, *P*-value**	**HR, 95% CI, *P*-value**	**HR, 95% CI, *P*-value**	**HR, 95% CI, *P*-value**
**Cirrhosis**			**Unadjusted**	Adjusted*	Adjusted**	Adjusted***
Continuous	2,567	428 (16.7)	1.10 (1.09–1.11), <0.001	1.11 (1.10–1.12), <0.001	1.09 (1.08–1.10), <0.001	1.06 (1.04–1.07), <0.001
**Categorical**						
[0~1.2)	468	23 (4.9)	1 (Reference)	1 (Reference)	1 (Reference)	1 (Reference)
[1.2~1.5)	852	65 (7.6)	1.49 (0.93–2.39), 0.102	1.53 (0.95–2.46), 0.082	1.34 (0.86–2.24), 0.18	1.27 (0.79–2.05), 0.324
[1.5~2.0)	724	105 (14.5)	2.79 (1.77–4.38), <0.001	2.97 (1.88–4.67), <0.001	2.53 (1.60–3.99), <0.001	1.82 (1.14–2.90), 0.011
[2.0~2.5)	275	98 (35.6)	7.94 (5.04–12.51), <0.001	8.99 (5.68–14.22), <0.001	7.39 (4.64–11.76), <0.001	3.78 (2.30–6.07), <0.001
[2.5, ~)	248	137 (55.2)	13.57 (8.72–21.11), <0.001	15.70 (10.03–24.58), <0.001	10.69 (6.72–17.00), <0.001	4.45 (2.72–7.28), <0.001
**Advanced fibrosis**			**Unadjusted**	Adjusted*	**Adjusted** ^Δ^	**Adjusted** ^ΔΔ^
Continuous	924	69 (7.5)	1.14 (1.12–1.16), <0.001	1.14 (1.12–1.16), <0.001	1.11 (1.08–1.14), <0.001	1.09 (1.06–1.12), <0.001
**Categorical**						
[0~1.5)	626	5 (0.8)	1 (Reference)	1 (Reference)	1 (Reference)	1 (Reference)
[1.5~2.0)	165	12 (7.3)	9.81 (3.46–27.85), <0.001	10.33 (3.60–29.65), <0.001	8.13 (2.81–23.53), <0.001	6.35 (2.15–18.71), <0.001
[2.0~2.5)	75	23 (30.7)	43.20 (16.42–113.66), <0.001	51.28 (18.96–138.75), <0.001	34.03 (12.27–94.41), <0.001	22.40 (7.70–65.16), <0.001
[2.5, ~)	58	29 (50)	72.04 (27.85–186.33), <0.001	75.75 (28.86–198.85), <0.001	35.50 (12.18–103.50), <0.001	23.84 (7.88–72.16), <0.001

Adjusted*, adjusted age, gender, etiology; Adjusted**, adjusted age, gender, etiology, HE grades, ascites, infection, gastrointestinal bleeding; Adjusted***, adjusted age, gender, etiology, HE grades, ascites, infection, gastrointestinal bleeding, TB and Cr; Adjusted^Δ^, adjusted age, gender, etiology, HE grades, ascites, infection; Adjusted^ΔΔ^, adjusted age, gender, etiology, HE grades, ascites, infection, TB and Cr.

### INR = 1.6 and 1.7 Are the Starting Points of INR for Acute Disease Deterioration in Cirrhosis and Advanced Fibrosis, Respectively

**Figure 5:**
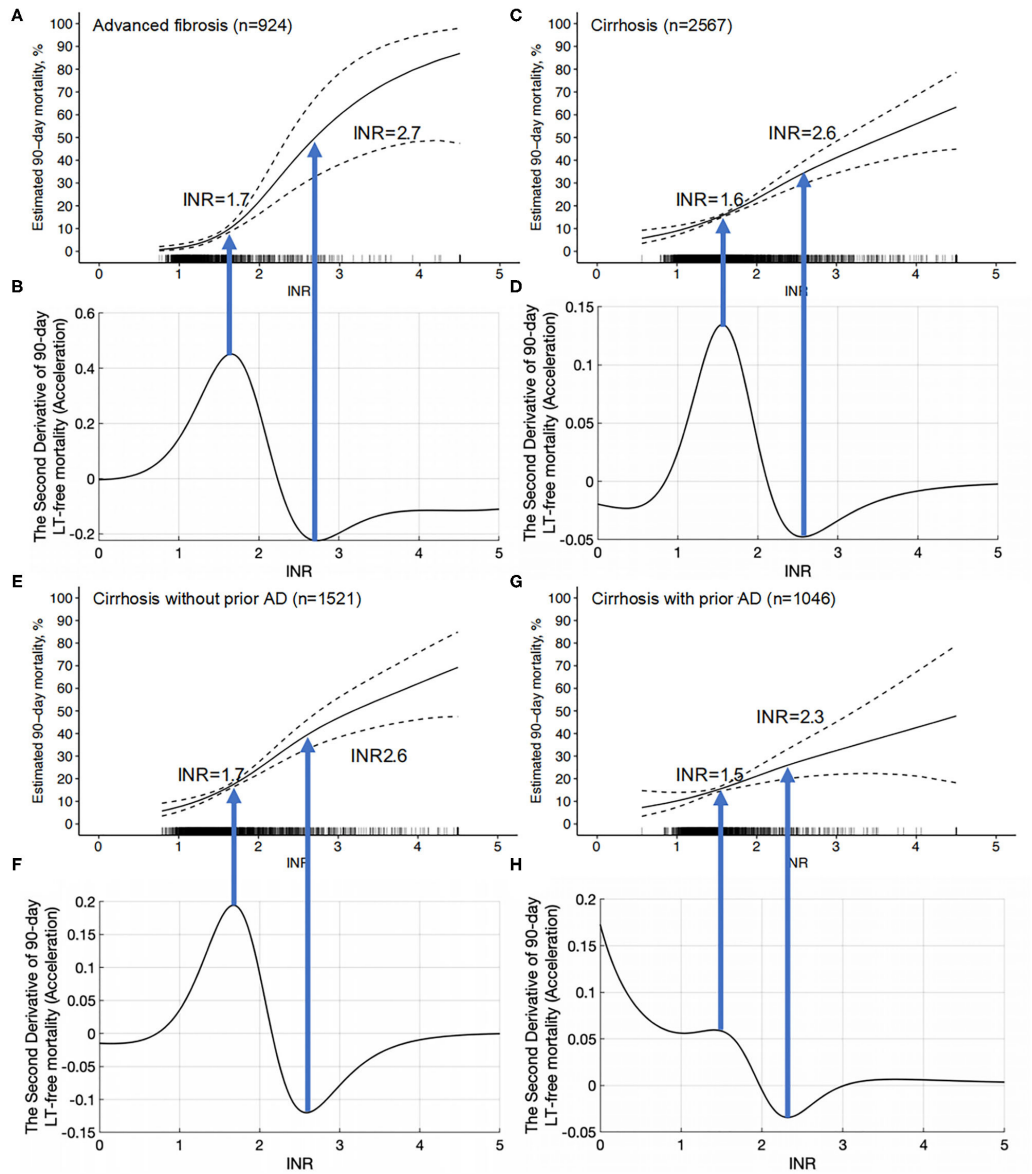
The INR-mortality (90-day) correlation curves and their corresponding second derivative (acceleration) curves. **(A)** The INR-mortality (90-day) correlation curve of advanced fibrosis, **(B)** the second derivative (acceleration) of INR to mortality in advanced fibrosis, **(C)** the INR-mortality (90-day) correlation curve of cirrhosis, **(D)** the second derivative (acceleration) of INR to mortality in cirrhosis, **(E)** the INR-mortality (90-day) correlation curve of cirrhosis without prior AD, **(F)** the second derivative (acceleration) of INR to mortality in cirrhosis without prior AD, **(G)** the INR-mortality (90-day) correlation curve of cirrhosis with prior AD, and **(H)** the second derivative (acceleration) of INR to mortality in cirrhosis with prior AD.

Through the peak of the second derivative, we found that the maximum acceleration point of the INR (INR = 1.6) corresponded to the most rapid increase in mortality on the GAM curve, which independently reflected the relationship between the INR and 90-day LT-free mortality (Figures 5C,D). The valley of the second derivative was when the INR = 2.6, which showed an INR of 1.6~2.6 is the period with the most rapid increase in mortality. This suggests that an INR = 1.6 is the starting point of the INR for disease deterioration in cirrhosis. We further analyzed the relationship between the INR and 90-day LT-free mortality in patients with cirrhosis without prior AD and patients with cirrhosis with any prior AD. The trends of the two curves were similar, with starting points of INR = 1.7 and 1.5 for disease deterioration, respectively, which were similar to the values in patients with cirrhosis (Figures 5E–H).

In patients with advanced fibrosis, the peak INR value of the second derivative was 1.7, and the valley INR value was 2.7, which indicates that mortality rises the fastest when the INR is between 1.7 and 2.7. Therefore, an INR value of 1.7 can be used as the starting point of INR for disease deterioration (Figures 5A,B).

### INR = 2.1 Was Associated With 28-Day LT-Free Mortality of 15% Both in Patients With Cirrhosis and in Patients With Advanced Fibrosis

**Figure 6:**
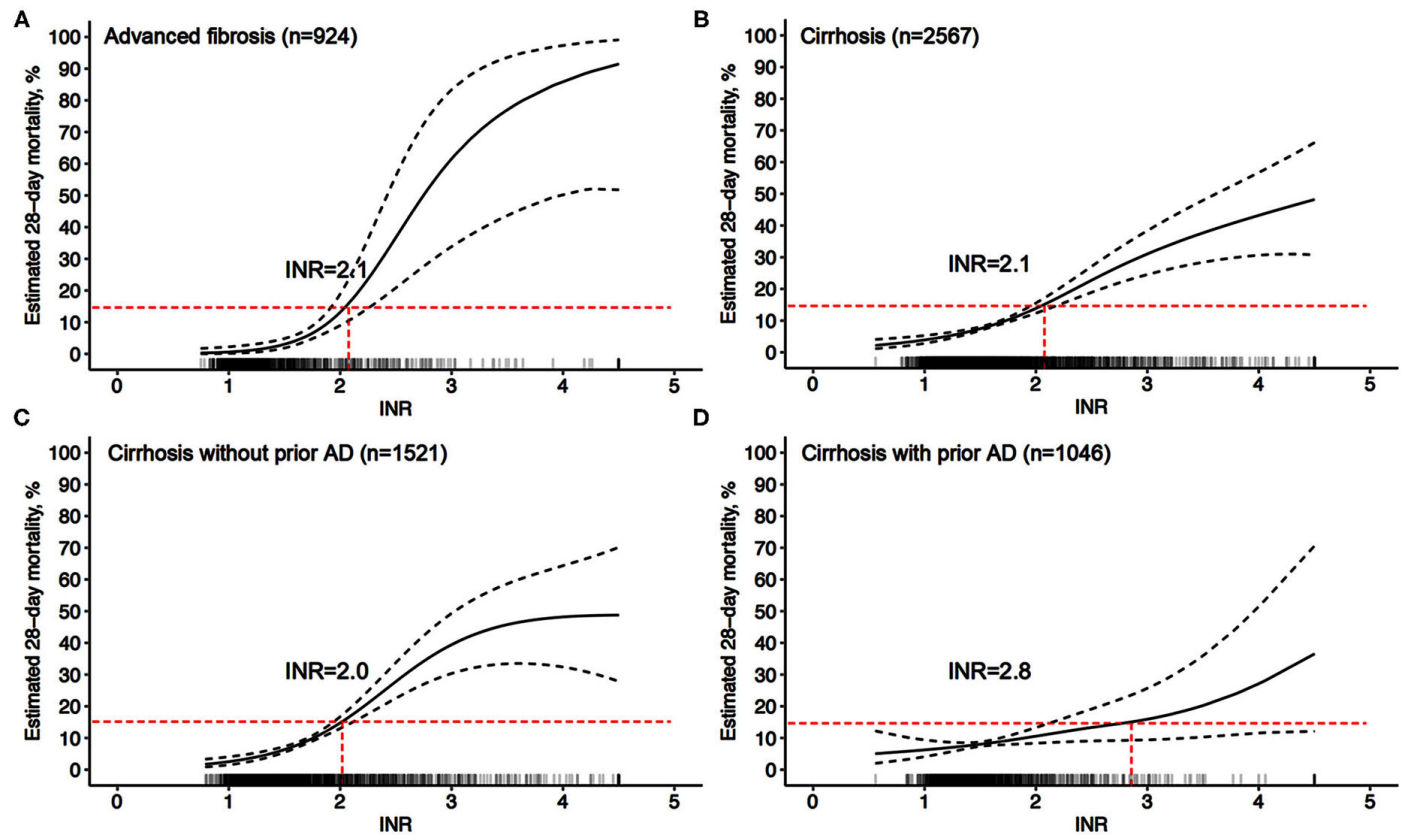
Adjusted probability of INR-mortality (28-day) correlation curves. **(A)** adjusted (age, gender, etiology, HE grades, ascites, infection, TB, and Cr) probability of the INR-mortality (28-day) correlation curve in advanced fibrosis, **(B–D)** adjusted (age, gender, etiology, HE grades, ascites, infection, gastrointestinal bleeding, TB, and Cr) probability of the INR-mortality (28-day) correlation curve in cirrhosis **(B)**, in cirrhosis without prior AD **(C)**, and in cirrhosis with prior AD **(D)**.

EASL defines organ failure as a 28-day LT-free mortality of 15%; therefore, the quantitative analysis found that an INR = 2.1 corresponded to an LT-free mortality of 15% within 28 days after admission in cirrhotic patients (Figure 6). An INR = 2.1 can be used as the clinical cutoff value for coagulation failure in patients with liver cirrhosis, with the corresponding multivariable adjusted 28/90-day LT-free mortality of 15 and 25.1%, respectively (Table 4), and 447 (17.4%) patients in our study exceeded this clinical cutoff of INR for coagulation failure. Similarly, in patients with advanced fibrosis, an INR = 2.1 was also found to correspond to a 28-day LT-free mortality of 15%, and 116 (12.6%) patients in our study exceeded this threshold, with corresponding 28/90-day LT-free mortality of 15 and 26.3%, respectively. Therefore, these values can be used as the clinical cutoff values of INR for coagulation failure.

**Table 4:** Multivariable adjusted 28- and 90-day LT-free mortality based on the starting point of INR for disease deterioration and clinical INR cutoffs in cirrhosis and advanced fibrosis.

	**Meaning of**	**Value of**	**Number of patients**	**28-day LT-free**	**90-day LT-free**
	**INR cutoff**	**cutoff**	**exceeding cutoff (%)**	**mortality (%)**	**mortality (%)**
Cirrhosis	Peak of acceleration curve	1.6	40.2% (1033/2567)	8.5%	16.2%
	Valley of acceleration curve	2.6	8.4% (215/2567)	24.7%	34.7%
	Reaching 15% 28-day LT-free mortality (clinical cutoff)	2.1	17.4% (447/2567)	15%	25.1%
	Coagulation dysfunction without coagulation failure	1.6–2.1	22.8% (586/2567)	7.8%	17.4%
Advanced fibrosis	Peak of acceleration curve	1.7	22.3% (206/924)	5.6%	11.9%
	Valley of acceleration curve	2.7	5.6% (52/924)	46.5%	49.7%
	Reaching 15% 28-day LT-free mortality (clinical cutoff)	2.1	12.6% (116/924)	15%	26.3%
	Coagulation dysfunction without coagulation failure	1.7–2.1	9.7% (90/924)	3.3%	7.8%

The short-term LT-free mortality in patients with INR values between the starting point for disease deterioration and cutoff for coagulation failure was further analyzed. An INR of 1.6~2.1 in the patients with cirrhosis was corresponded to 28/90-day LT-free mortality of 7.8 and 17.4%, respectively. Similarly, the INR value of 1.7~2.1 in patients with advanced fibrosis was associated with a 28/90-day LT-free mortality of 3.3 and 7.8%, respectively (Table 4).

## Discussion

This study quantitatively described in detail the relationship between INR values and short-term LT-free mortality for the first time in patients with cirrhosis or advanced fibrosis through a multicenter, prospective cohort study. First, the results showed that the INR value was an independent risk factor for both 28- and 90-day LT-free mortality in patients with either cirrhosis or advanced liver fibrosis. In both patients with cirrhosis and in patients with advanced fibrosis, an INR > 1.5 had a more significant impact on 90-day LT-free mortality than an INR <1.5. Second, we found for the first time the starting point of the INR that indicates acute disease deterioration (coagulation dysfunction) in a highly endemic area of HBV. Finally, when the INR = 2.1, the 28-day LT-free mortality reached 15% in both patients with cirrhosis and patients with advanced fibrosis. Therefore, an INR > 2.0 could be used as the cutoff of INR for the diagnosis of coagulation failure in either patients with cirrhosis or advanced fibrosis.

In the diagnostic criteria of ACLF, the study population of the APASL included patients with cirrhosis and noncirrhotic chronic liver diseases, while the study population of the EASL included patients with cirrhosis only (7, 9, 25). To resolve this controversy, the World Gastroenterology Organization (WGO) proposed that significant hepatic fibrosis can be considered chronic hepatitis to help distinguish ACLF from acute liver failure (26–28). An FIB-4 > 1.45 was used to screen advanced fibrosis in noncirrhotic chronic liver diseases (21). In the present study, in patients with advanced fibrosis, the INR corresponding to a 28-day LT-free mortality of 15% was also 2.1, and this result suggested that coagulation failure in patients with advanced fibrosis shares the same INR cutoff value, which is also consistent with patients with cirrhosis. Furthermore, in patients with cirrhosis and advanced fibrosis who presented coagulation dysfunction before coagulation failure (INR = 16–2.1), the 28-day LT-free mortality was much lower than 15%, providing more evidence that INR > 2.0 could be used as the cutoff of INR for the diagnosis of coagulation failure in either patients with cirrhosis or advanced fibrosis.

In this study, the INR was not an independent risk factor for 90-day LT-free mortality in subgroups HE3–4. HE is one of the common complications of ALI or AD in patients with cirrhosis and other chronic liver diseases (29–31). The North American Liver Failure Alliance (NACSELD) identified HE grades 3–4 as an independent predictor of 30-day mortality in hospitalized patients with cirrhosis, with 30-day mortality for HE grades 3–4 as high as 38% (32, 33). Our previous study also demonstrated that the 28-day LT-free mortality rates were 34.5 and 55% for HE grades 3 and 4, respectively, among patients with cirrhosis and other chronic liver diseases (34). The results from the above studies and the results from our study demonstrated that grade 3–4 HE plays a more important role than the INR in the short-term outcomes of patients with cirrhosis.

Our research has the following strengths. First, the data come from a high-quality large-scale prospective cohort in a high-endemic area of HBV (17, 18, 20). Second, we obtained for the first time the evidence-based starting point of the INR value that indicates acute disease deterioration both in patients with cirrhosis and in patients with advanced fibrosis, and this can be used as a warning sign of a rapid increase in mortality. The starting point of the INR is important for clinicians to timely identify the rapid increase in mortality caused by coagulation damage. Finally, the unified clinical cutoff of the INR for coagulation failure was found in patients with cirrhosis and advanced fibrosis.

This study had several limitations. First, the data in this study came from HBV high-endemic areas that were not representative of the global characteristics of patients with cirrhosis and other chronic liver diseases, but HBV accounts for 70% of the cases of cirrhosis and other chronic liver diseases and is the highlight of our cohort study. Second, our study only included LT-free mortality as the outcome and did not include liver transplantation as a bad outcome. In most major studies, LT-free mortality was used as the only end point, which is consistent with our study (7, 35, 36). Finally, this was an observational study that did not investigate the effects of special treatments (such as artificial liver support systems, glucocorticoids, etc.) on the short-term adverse outcomes of patients with cirrhosis and advanced fibrosis. To date, no treatment except liver transplantation has been shown to significantly change the outcome of ACLF.

This was the first study to quantitatively describe the relationship between the INR and short-term LT-free mortality in patients with cirrhosis and advanced fibrosis. For the first time, we obtained the starting point of the INR that indicated a rapid increase in mortality. Patients with either cirrhosis or advanced fibrosis share the same clinical cutoff INR value for the diagnosis of coagulation failure.

## Data Availability Statement

The original contributions presented in the study are included in the article/Supplementary Materials, further inquiries can be directed to the corresponding author/s.

## Ethics Statement

The studies involving human participants were reviewed and approved by the Medical Ethics Board of Shanghai Renji Hospital and Shiyan Taihe Hospital. The patients/participants provided their written informed consent to participate in this study.

## Author Contributions

HL, SS, XW, XinZ, YaH, BL, ZM, YG, ZQ, FL, XL, JuL, HR, GD, YuZ, and HY contributed to the conception and design of the study. HL, XW, XinZ, YaH, ZM, YG, ZQ, FL, XL, JuL, GD, YuZ, HY, LQ, YaZ, WG, XX, YiZ, SS, BX, YiH, QZ, YX, CZ, JC, ZH, BL, XJ, TQ, SL, YC, NG, CL, WY, XM, JiL, TL, RZ, XinyiZ, and HR contributed to organization and data collection. YW wrote the first draft of the manuscript. FD and WZ performed the statistical analysis. ZM and HL performed critical revision of the manuscript for important intellectual content. All authors read and approved the final version of the submitted manuscript.

## Funding

This research was supported by the National Science and Technology Major Project (2018ZX10723203, 2018ZX10302206, and 2017ZX10202202), National Natural Science Foundation of China (82070650, 81930061), Shanghai Hospital Development Commission (16CR1024B and SHDC2020CR1037B), Shanghai Municipal Education Commission–Guofeng Clinical Medicine and Shanghai Municipal Government Funding, National Key Research and Development Program of China (2017YFC0908100), the Foundation for Innovative Research Groups of Hubei Provincial Natural Science Foundation (2018CFA031), Hubei Province's Outstanding Medical Academic Leader Program, and Project of Hubei University of Medicine (FDFR201902, 2020XGFYZR05, 2021ESOF011, and YC2021012).

## Conflict of Interest

The authors declare that the research was conducted in the absence of any commercial or financial relationships that could be construed as a potential conflict of interest. The reviewer ZC declared a shared parent affiliation with the authors HL, FD, LQ, YZha, WG, and WZ and the reviewer QY declared a shared parent affiliation with the author HR to the handling editor at the time of the review.

## Publisher's Note

All claims expressed in this article are solely those of the authors and do not necessarily represent those of their affiliated organizations, or those of the publisher, the editors and the reviewers. Any product that may be evaluated in this article, or claim that may be made by its manufacturer, is not guaranteed or endorsed by the publisher.
