# Supplementary material for: Increased INR Values Predict Accelerating Deterioration and High Short-Term Mortality Among Patients Hospitalized With Cirrhosis or Advanced Fibrosis
Source: Front Med (Lausanne). 2021 Nov 18;8:762291. doi: 10.3389/fmed.2021.762291 (PMC8637055; doi:10.3389/fmed.2021.762291)
Supplement: Supplementary file 1 [file Table_1.DOCX]

Table S1. The unadjusted and adjusted hazard ratios of INR for 28-day transplantation-free mortality in patients with cirrhosis and advanced fibrosis.

| INR | n | Death (%) | HR, 95% CI, P-value | HR, 95% CI, P-value | HR, 95% CI, P-value | HR, 95% CI, P-value |
| --- | --- | --- | --- | --- | --- | --- |
| Cirrhosis |  |  | **Unadjusted** | **Adjusted*** | **Adjusted**** | **Adjusted***** |
| continuous | 2567 | 236(9.2) | 1.113(1.1-1.126), <0.001 | 1.117(1.104-1.13), <0.001 | 1.103(1.089-1.117), <0.001 | 1.074(1.059-1.09), <0.001 |
| categorical |  |  |  |  |  |  |
| [0~1.2) | 468 | 10(2.1) | 1(Reference) | 1(Reference) | 1(Reference) | 1(Reference) |
| [1.2~1.5) | 852 | 29(3.4) | 1.561(0.761-3.203), 0.225 | 1.624(0.79-3.336), 0.187 | 1.471(0.715-3.029), 0.294 | 1.379(0.669-2.842), 0.384 |
| [1.5~2.0) | 724 | 44(6.1) | 2.729(1.373-5.422), 0.004 | 3.011(1.509-6.005), 0.002 | 2.619(1.307-5.252), 0.007 | 2.016(0.997-4.078), 0.051 |
| [2.0~2.5) | 275 | 55(20) | 9.826(5.009-19.276), <0.001 | 11.724(5.938-23.15), <0.001 | 10.146(5.087-20.236), <0.001 | 5.792(2.836-11.829), <0.001 |
| [2.5, ~) | 248 | 98(39.5) | 21.534(11.231-41.286), <0.001 | 26.206(13.556-50.663), <0.001 | 18.615(9.424-36.771), <0.001 | 8.947(4.373-18.304), <0.001 |
| Advanced fibrosis |  |  | **Unadjusted** | **Adjusted*** | **Adjusted**△ | **Adjusted**△△ |
| continuous | 924 | 40(4.3) | 1.17(1.14-1.2), <0.001 | 1.17(1.14-1.2), <0.001 | 1.14(1.1-1.17), <0.001 | 1.12(1.07-1.16), <0.001 |
| categorical |  |  |  |  |  |  |
| [0~1.5) | 384 | 0(0) | 1(Reference) | 1(Reference) | 1(Reference) | 1(Reference) |
| [1.5~2.0) | 242 | 1(0.4) | 15.65(1.75-139.99), 0.01 | 15.36(1.71-137.55), 0.01 | 12.72(1.41-114.8), 0.02 | 8.47(0.92-77.72), 0.06 |
| [2.0~2.5) | 165 | 4(2.4) | 97.75(12.62-757.15), <0.001 | 98.09(12.6-763.78), <0.001 | 74.98(9.48-593.23), <0.001 | 38.39(4.66-316.3), <0.001 |
| [2.5, ~) | 75 | 11(14.7) | 296.38(40.07-2192.03), <0.001 | 283.4(38.13-2106.55), <0.001 | 125.82(15.76-1004.8), <0.001 | 69.04(8.38-568.83), <0.001 |

Adjusted*, adjusted age, gender, etiology; Adjusted**, adjusted age, gender, etiology, HE grades, ascites, infection, gastrointestinal bleeding; Adjusted***, adjusted age, gender, etiology, HE grades, ascites, infection, gastrointestinal bleeding, TB and Cr; Adjusted△, adjusted age, gender, etiology, HE grades, ascites, infection; Adjusted△△, adjusted age, gender, etiology, HE grades, ascites, infection, TB and Cr.

Table S2. Multivariable adjusted 28-day and 90-day LT-free mortality based on the starting point of INR for disease deterioration and clinical INR cutoffs in cirrhosis and advanced fibrosis with HBV or non-HBV.

|  | Meaning of INR cutoff | Etiology | Value of  cutoff | Num of patients  exceeding cutoff (%) | 28-day LT-free  mortality (%) | 90-day LT-free  mortality (%) |
| --- | --- | --- | --- | --- | --- | --- |
| Cirrhosis | Peak of acceleration curve | Total | 1.6 | 40.2% (1033/2567) | 8.5% | 16.2% |
|  |  | HBV | 1.5 | 53.9% (922/1712) | 6.9% | 14.3% |
|  |  | Non-HBV | 1.8 | 17.9% (153/855) | 9.4% | 17.9% |
|  | Valley of acceleration curve | Total | 2.6 | 8.4% (215/2567) | 24.7% | 34.7% |
|  |  | HBV | 2.5 | 11.7% (198/1712) | 23.7% | 33.8% |
|  |  | Non-HBV | 2.6 | 4.1% (35/855) | 18.0% | 29.1% |
|  | Reaching 15% 28-day  LT-free mortality (clinical cutoff) | Total | 2.1 | 17.4% (447/2567) | 15% | 25.1% |
|  |  | HBV | 2.0 | 24.1% (417/1712) | 15% | 23.6% |
|  |  | Non-HBV | 2.3 | 7.4% (63/855) | 15.0% | 25.1% |
| Advanced  fibrosis | Peak of acceleration curve | Total | 1.7 | 22.3% (206/924) | 5.6% | 11.9% |
|  |  | HBV | 1.6 | 28.8% (222/770) | 4.2% | 9.0% |
|  |  | Non-HBV | - | - | - | - |
|  | Valley of acceleration curve | Total | 2.7 | 5.6% (52/924) | 46.5% | 49.7% |
|  |  | HBV | 3.0 | 0.3% (22/770) | 62.1% | 57.3% |
|  |  | Non-HBV | - | - | - | - |
|  | Reaching 15% 28-day  LT-free mortality (clinical cutoff) | Total | 2.1 | 12.6% (116/924) | 15% | 26.3% |
|  |  | HBV | 2.1 | 14.0% (108/770) | 15% | 24.3% |
|  |  | Non-HBV | - | - | - | - |

-, lacking of data.

**Fig. S1. Pattern of missing data.** Abbreviations: HE, hepatic encephalopathy; GI bleeding, gastrointestinal bleeding; INR, international normalized ratio; TB, total bilirubin; Cr, creatinine.

**Fig. S2. The INR-mortality (90-day) correlation curves and their corresponding second derivative (acceleration) curves in patients with HBV or non-HBV**. A, the INR-mortality (90-day) correlation curve of cirrhosis with HBV; B, the second derivative (acceleration) of INR to mortality in cirrhosis with HBV; C, the INR-mortality (90-day) correlation curve of cirrhosis with non-HBV; D, the second derivative (acceleration) of INR to mortality in cirrhosis with non-HBV; E, the INR-mortality (90-day) correlation curve of advanced fibrosis with HBV; F, the second derivative (acceleration) of INR to mortality in advanced fibrosis with HBV; G, the INR-mortality (90-day) correlation curve of advanced fibrosis with non-HBV.

**Fig. S3. Adjusted probability of** **INR-mortality (28-day) correlation curves in patients with HBV or non-HBV**. A-B, adjusted (age, gender, etiology, HE grades, ascites, infection, gastrointestinal bleeding, TB and Cr) probability of the INR-mortality (28-day) correlation curve in cirrhosis with HBV (A) and cirrhosis with non-HBV. C-D, adjusted (age, gender, etiology, HE grades, ascites, infection, TB and Cr) probability of the INR-mortality (28-day) correlation curve in advanced fibrosis with HBV and advanced fibrosis with non-HBV.
