# Supplementary figures and images for: Increased INR Values Predict Accelerating Deterioration and High Short-Term Mortality Among Patients Hospitalized With Cirrhosis or Advanced Fibrosis
Source: Front Med (Lausanne). 2021 Nov 18;8:762291. doi: 10.3389/fmed.2021.762291 (PMC8637055; doi:10.3389/fmed.2021.762291)

## Slide 1
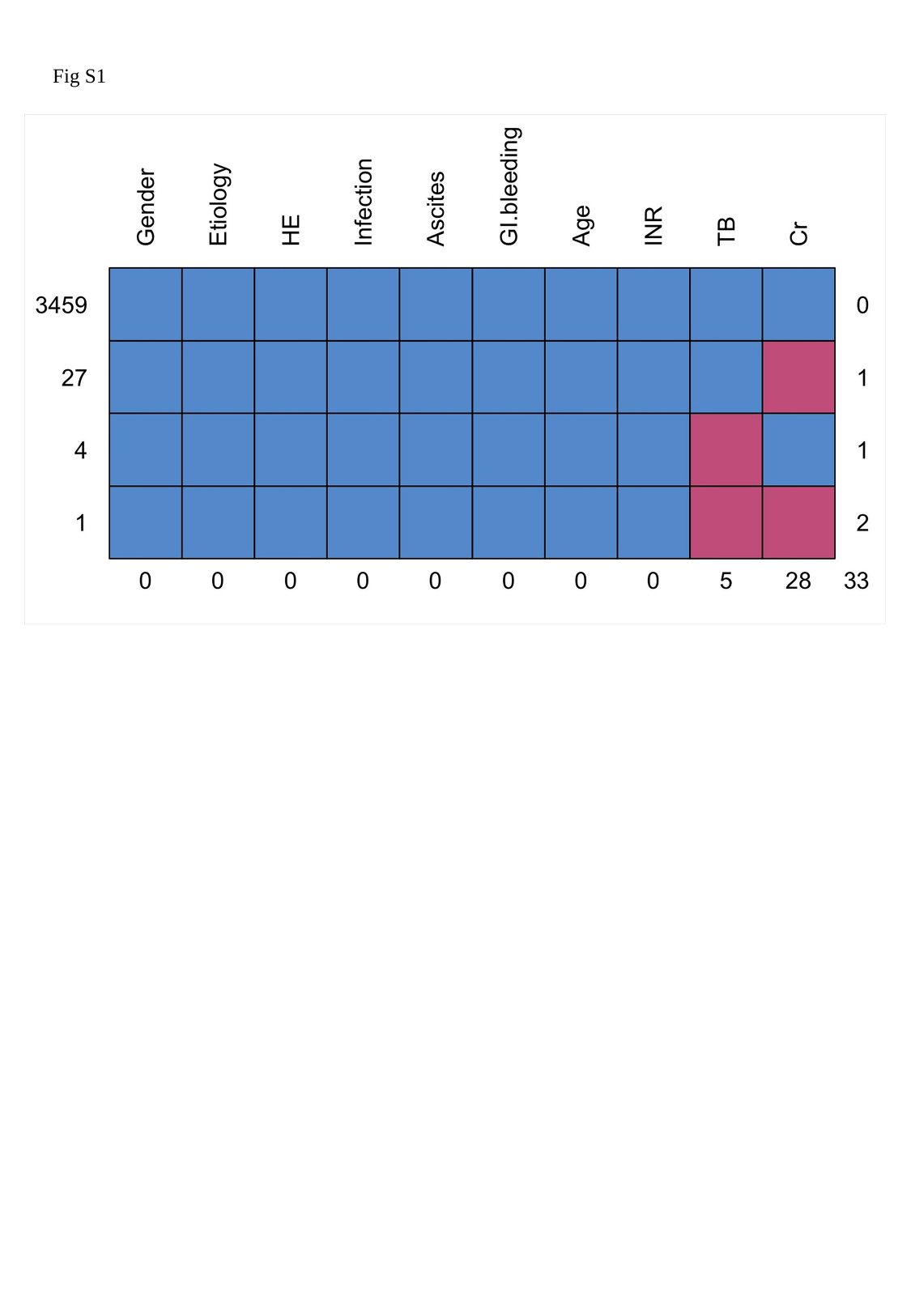

Fig S1

## Slide 2
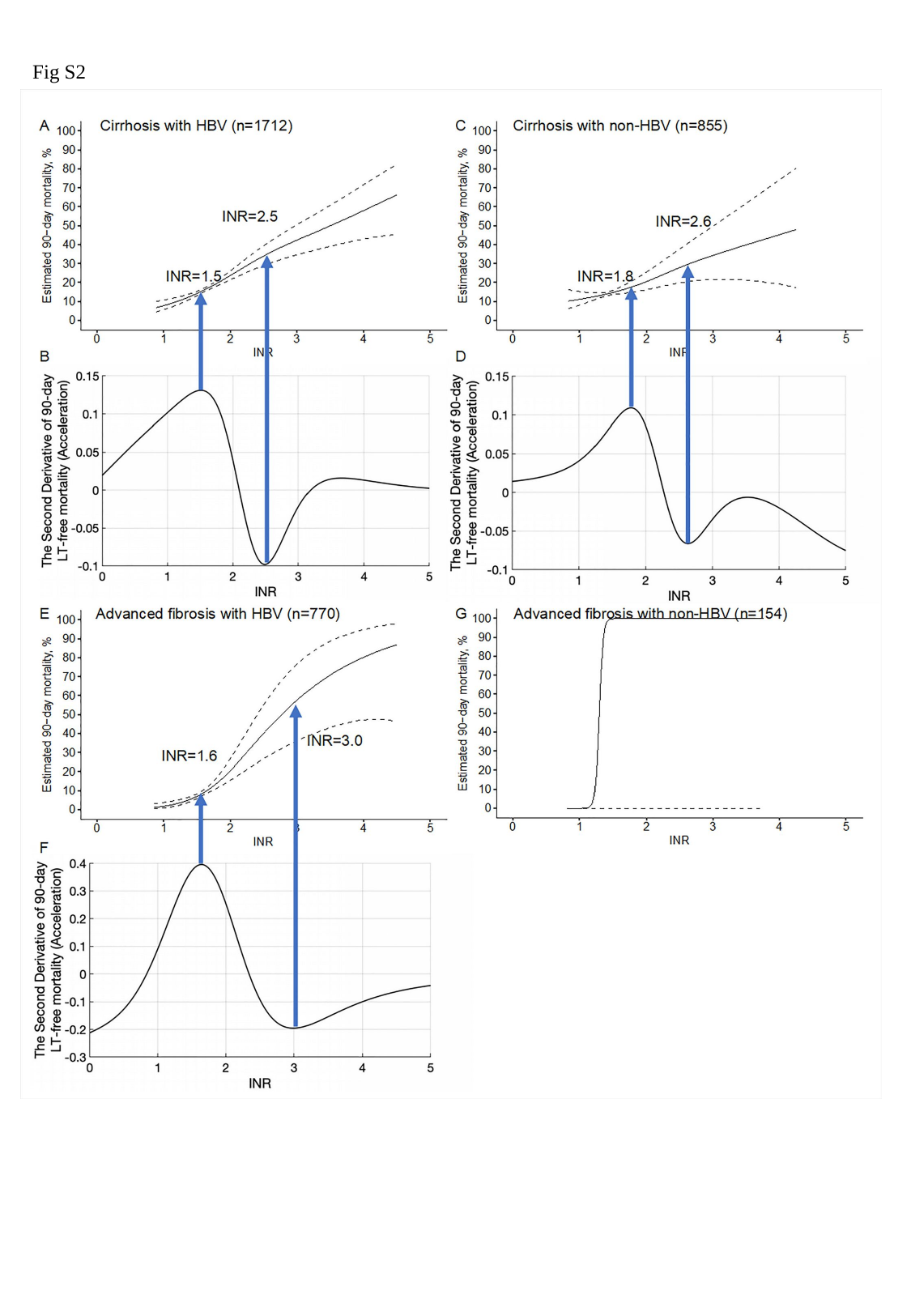

Fig S2

## Slide 3
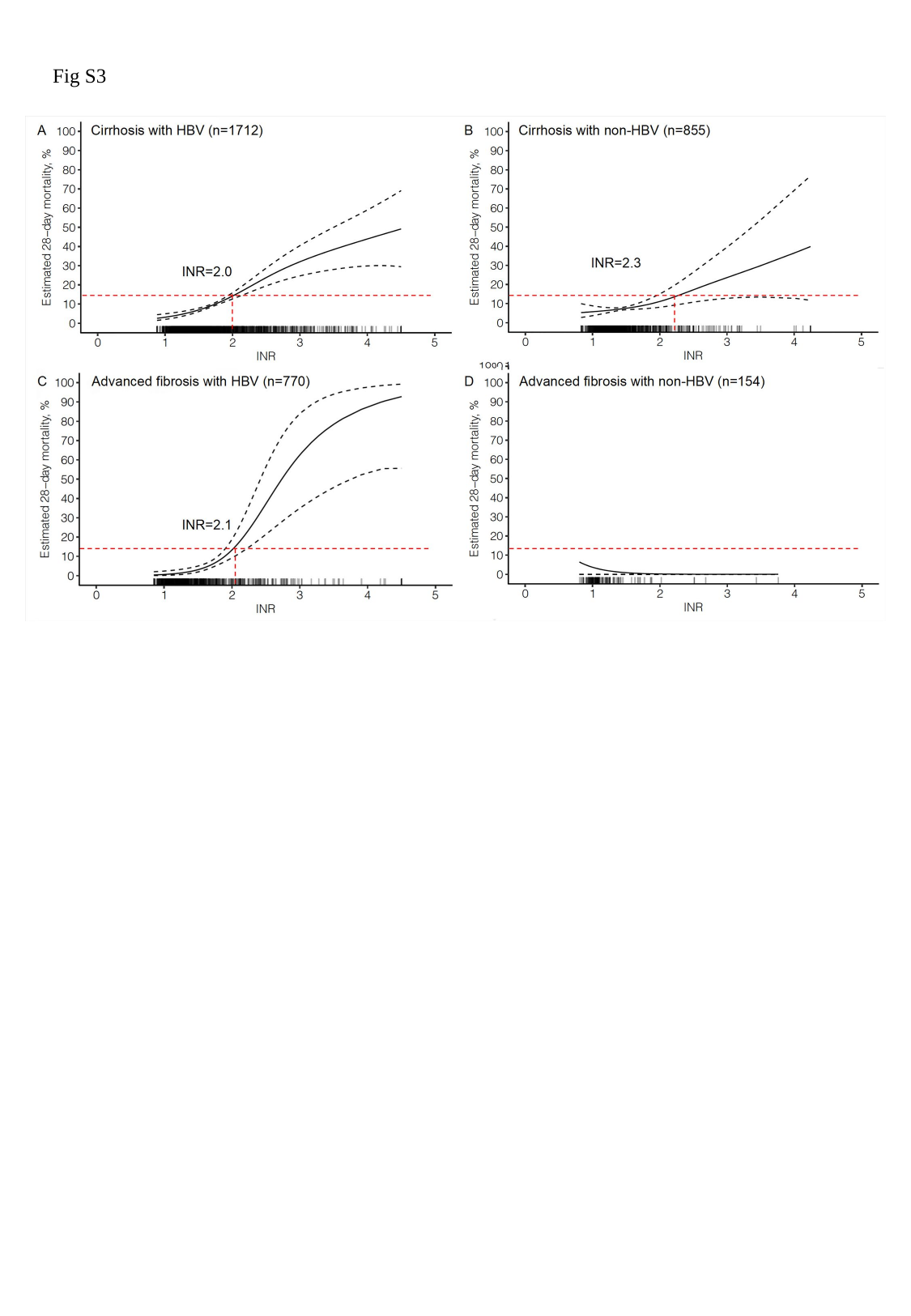

Fig S3

Supplement: Supplementary file 2 [file Presentation_1.PPTX]
